# Considerations of temperature in the context of the persistence classification in the EU

**DOI:** 10.1186/s12302-017-0113-1

**Published:** 2017-04-05

**Authors:** Michael Matthies, Sabine Beulke

**Affiliations:** 1 Institute of Environmental Systems Research (USF), University of Osnabrück, 49069 Osnabrück, Germany; 2 Enviresearch Ltd, 34 Grainger Park Road, Newcastle upon Tyne, NE4 8RY UK

**Keywords:** PBT assessment, Exposure assessment, Environmental temperature, Hazard, Degradation simulation testing, Persistence criteria, Arrhenius equation, Temperature normalisation

## Abstract

Simulation degradation studies for industrial chemicals, biocidal products and plant protection products are required in the EU to estimate half-lives in soil, water and sediment for the comparison to persistence criteria for hazard (P/vP) assessment, and for use in exposure assessments. There is a discrepancy between European regulatory approaches regarding the temperature at which degradation half-lives should be (1) measured in simulation degradation testing of environmental compartments, and (2) compared to the P/vP criteria. In this paper, an opinion is provided on the options for the experimental temperature and extrapolation to other conditions. A review of the historical development of persistence criteria did not give conclusive evidence of the temperature at which the half-lives that underpin the P-criteria were measured, but room temperature is likely. Half-lives measured at 20 °C are in line with the intentions of some international agreements, but in the EU there is a continued political debate regarding the relevant temperature for comparison with persistence criteria. Measuring degradation at 20 °C has the advantage that metabolites/transformation products can be identified with greater accuracy, and that kinetic fits to determine half-lives for parent compounds and metabolites carry less uncertainty. Extrapolation of half-lives to lower temperatures is possible for assessing environmental exposure, but the uncertainty of the persistence classification is smaller when measured half-lives are used for direct comparison with P/vP criteria, without extrapolation. Model simulations demonstrate the pattern of concentrations that can be expected for *realistic worst case* climate scenarios in the EU based on the half-life of 120 days in soil at 20 °C and of 40 days in water at 20 °C, and their temporal and spatial variability.

## Background

Classification of a chemical as being persistent (P), bioaccumulative (B) and toxic (T) or very persistent (vP) and very bioaccumulative (vB) is an integral part of the chemical legislation in the European Union (EU). PBT/vPvB substances can give rise to specific concerns due to their potential to accumulate in parts of the environment, which is in practice difficult to reverse and the effects of such accumulation are unpredictable in the long-term [15]. Criteria for PBT/vPvB hazard assessment are laid down in the European regulation for industrial chemicals registered under REACH [23], biocidal products [25], plant protection products (PPP) [24], and veterinary medicines [21]. These criteria are identical across European regulatory frameworks but the consequences are quite different [36]. Guidance for PBT/vPvB assessment of industrial chemicals and biocidal products has been provided since 2003 in Technical Guidance Documents and their revisions [12, 13, 15, 17, 26]. Although chlorinated PPPs have been amongst the first acknowledged POPs (persistent organic pollutants), PBT/vPvB assessment of PPP was only introduced in 2009 with the new regulation EC 1107/2009 [24], but no guidance was provided on how to conduct a PBT/vPvB assessment [46]. A working document was issued by DG [10] which laid down the evidence needed to identify POP, PBT and vPvB properties for PPPs.

For persistence classification in the EU, degradation half-life criteria have been defined for fresh, estuarine and marine waters and sediments and for soil (Table 1). However, it is not clear what the relevant conditions are for the half-lives to be compared with persistence criteria. In particular, temperature has a great influence on the degradation half-lives in water, sediment and soil since (bio)chemical reactions are temperature dependent. For PPP, a reference temperature of 20 °C is recommended and degradation half-lives^1^ should be normalised to this temperature if the study has been undertaken at a different temperature [10]. On the other hand, a temperature of 12 °C as a standard environmental characteristic and 9 °C for abiotic marine degradation has been proposed for industrial chemicals and biocidal products [15, 16, 26]. Moreover, the temperature at which chemicals should be tested in laboratory simulation studies is still a matter of discussion and is related to the method of extrapolation to other temperatures. Thus, there is a discrepancy between the various European regulatory approaches regarding the temperature at which degradation half-lives should be [1] tested in simulating degradation testing of environmental compartments, and [2] compared to the P/vP criteria of Table 1.

**Table 1:** Criteria for the classification as P or vP [23]

Criteria for persistence (P)	Criteria for very persistent (vP)
Marine water: *t*½ > 60 days Fresh and estuarine water: *t*½ > 40 days Marine sediment: *t*½ > 180 days Fresh and estuarine sediment: *t*½ > 120 days Soil: *t*½ > 120 days	Water: *t*½ > 60 days Sediment: *t*½ > 180 days Soil: *t*½ > 180 days

The objectives of the paper are to provide an opinion on the following:

- The temperature at which the half-life should be measured for P/vP assessment.
- How the half-life should be derived (measure at 20 °C or measure at 12 °C and then extrapolate to 20 °C).
- How the extrapolation should be undertaken and under which conditions it is valid.

## Environmental exposure and hazard (PBT/vPvB) assessment in the EU

### Exposure assessment

Degradation half-lives in water, sediment and soil are not only required as hazard properties in PBT/vPvB assessment, but also as input data for environmental and indirect human exposure and risk assessment. For this purpose, specific exposure scenarios and environmental transport and fate models have been developed and used for the calculation of predicted environmental concentrations (PECs) in air, water, soil and sediment. The environmental risk is characterised by dividing these PECs by appropriate PNECs (predicted no-effect concentrations) or by calculating Toxicity Exposure Ratios. For industrial chemicals and biocides, the Guidance on Information Requirements and Chemical Safety Chapter R.16 [14] details the methodology of environmental exposure estimation and the role of degradation half-lives. It is obvious that PECs should be calculated at the relevant temperature for the exposure models employed. A reference temperature of 12 °C is defined as average outdoor temperature in Europe and 9 °C as average temperature of marine waters.

However, degradation has only a limited impact on calculated PECs of industrial chemical and biocidal products. Degradation rate constants are input parameters for the calculation of PEC_regional_ and PEC_local_ for soil, but not for sediment and surface water, which is the receiving compartment for almost all industrial chemicals and biocides. Moreover, half-lives for biodegradation in surface water based on results of screening tests on biodegradability or (Q)SAR calculations do not need to be corrected for different environmental temperatures. Thus, temperature correction of degradation half-lives has only a minor influence on exposure and risk assessment for industrial chemicals and biocides.

For active substances of PPP, approved versions of FOCUS simulation models and FOCUS scenarios are used to calculate the concentrations in groundwater [27] and surface water [29] in the EU review process [24]. Degradation half-lives for soil, surface water and sediment are used as input parameters and these are adjusted to half-lives at the actual temperature on each day of the simulation period for the various European scenarios. Annual average temperatures range from 8 °C in northern to 18 °C in southern countries [27, 29]. Thus, the variability across the European climate zones is explicitly taken into account in the environmental exposure assessment, which makes an average European outdoor temperature obsolete for PPP. Regional distribution models are not part of exposure assessment of PPP.

### Hazard assessment

In chemical hazard assessment, intrinsic substance properties are directly compared to pre-defined criteria without any of the processing that is done in exposure models. These criteria are regarded as independent of the intended use, emission volume and mode of entry into the environment. Thus, there is a fundamental difference in environmental exposure and PBT/vPvB hazard assessment: (1) in environmental exposure assessment, concentrations are predicted by taking account of simultaneous processes of transport, distribution and degradation in and between representative environmental compartments at conditions relevant for the EU [15]; (2) in PBT/vPvB assessment, hazardous substance properties are compared to numerical and narrative criteria, which are based upon available data for reference chemicals, derived under laboratory conditions, and consensus-based policy discussions [1]. Thus, the scientific background as well as the political intentions and assumptions under which the criteria have been derived are of utmost significance.

## Reference temperature for persistence criteria in P/vP assessment

A brief summary of the historical development of persistence criteria is provided with particular consideration of temperature.

### Non-EU regulations and international agreements

Development of criteria for classification of persistent toxic substances goes back to the 70s of the last century. They evolved over the last decades due to the diversification of the protection aims under various national regulatory frameworks and international agreements [34]. Japan was the first nation regulating PCBs and other chloro-organic compounds by assessing their P, B, and T properties [35]. However, they did not define criteria for a formal assessment process. Canada and the USA recognised the need for elimination of persistent toxic substances from the Great Lakes and introduced the concept of half-life of a substance in water as a measure (criterion) for persistence [31, 50]: “Half-life means the time required for the concentration of a substance to diminish to one-half of its original value in a lake or water body”. They also defined a numerical value for the persistence criterion: “Persistent toxic substance means any toxic substance with a half-life in water of greater than eight weeks” [31, 50]. With the beginning of the 90s, various international commissions and North-American national institutions developed further the concept of environmental half-lives as persistence criteria and extended it to other environmental compartments (air, soil, sediment, ground water). An ad hoc science group of Environment Canada [22] reviewed degradation half-lives of a set of critical substances, mainly hydrophobic neutral organic compounds, such as PCBs, PAHs, PCDD/Fs. Scientific judgement was used to assign numerical values (half-lives) of 6 months in water and soil and 1 year in sediment, respectively, which became legally binding with the Canadian Environmental Protection Act [8]. The different half-lives reflect the persistence data available for the set of neutral hydrophobic reference chemicals, against which a chemical is to be compared. The group stated that “…under Canadian climate conditions 6 months is a reasonable window of time in which soil temperature and moisture favour biodegradation of many substances”.

With the evidence of long-range transboundary transport (LRT) of chemicals, broader international activities were launched for regulating persistent organic pollutants (POPs) [47, 49]. The specified half-life in water of 60 days was not based on new scientific findings or different environmental temperatures to be considered but was a compromise with a simultaneous decision for a bioconcentration factor (BCF) of 5000. The UNEP Criteria Expert group on POPs [48] stressed the need for the development and improvement of relevant test methods and recommended that the International Standards Organization (ISO) and the Organisation for Economic Cooperation and Development (OECD) undertook efforts to ensure that such needs concerning new and improved test methods were better met. Up to this point, temperature has not been explicitly considered for screening of chemicals based on their persistence according to the criteria in Annex D of the Stockholm Convention [49].

USEPA [51, 53] reviewed persistence criteria defined in various international agreements and national regulations and specified a lower boundary of 60 days for moderate action and an upper boundary of 180 days for high action without consideration of specific environmental compartments and conditions. A half-life of 60 days was chosen because the initial concentration of a released chemical drops to approximately 1% within 1 year, i.e. six half-lives. Soil biodegradation is tested at 22 ± 2 °C [52].

An international expert group at the SETAC Pellston workshop “Science-Based Guidance and Framework for the Evaluation and Identification of PBTs and POPs” stated that “It is reasonable to assume that persistence criteria, defined according to the reference chemical approach, are related to available half-life data for known POPs. Because these half-lives are likely to have been derived under laboratory conditions, these are the conditions for which the comparison should take place. According to the reference chemical approach, temperature correction, therefore, does not seem justified” [7].

### EU regulations

The Oslo Paris Convention (OSPAR) was the first European convention dealing with persistent substances in the environment [40, 41]. A half-life of 50 days in marine waters was defined as the only persistence criterion without further specification of the test methods and conditions, e.g. temperature. Around the year 2000, several papers and statements dealt with the temperature relevant for European climate conditions. The Swedish Committee on New Guidelines on Chemicals Policy [9] proposed that “a substance is regarded as unacceptably persistent if its half-life is longer than 8 weeks in a simulation test at 20 °C”. This half-life corresponds to a half-life of 1 year in the northern European climate (average annual temperature of 5 °C) and takes into account unfavourable degradation conditions. Sinkkonnen and Paassivirta [45] suggested degradation half-times for POPs as input parameters to the Baltic Sea model assuming an annual average temperature of 7 °C. Beyer et al. [5] could demonstrate that the LRT potential can monotonically increase or decrease with increasing temperature, or it can have a maximum in the temperature range between 5 and 30 °C.

The Technical Guidance Document (TGD) on Risk Assessment [26] added a new chapter on marine risk assessment, which also contains a subchapter on PBT assessment. Consequently, only criteria for water systems (fresh and marine waters and sediments) were defined (Table 1) in 2003, but not for soil. The numerical values for marine water (60 days) and sediment (180 days) as well as those for vP assessment are identical with those of international agreements, but for those fresh water (40 days) and fresh water sediment (120 days) are smaller. European outdoor temperature was not taken into consideration when defining the criteria.

PBT/vPvB assessment became legally binding with the release of REACH [23], which laid down the persistence criteria from the TGD [26] plus a half-life in soil of 120 days, which is shorter than in the international agreements. Again, the relevant temperature (and other environmental conditions) for testing persistence was not defined in the regulation. In the first version of the Guidance in Information and Chemical Safety Assessment Chapter R.11 [12], nothing is said on the relevant temperature for persistence testing. In version 2.0 of the Guidance Chapter R.11 [15], one sentence relates to temperature: “Please note that since its 32nd meeting the Member State Committee has started to require new simulation degradation studies to be carried out around neutral pH values and at 12 °C, which is understood as the mean temperature of European surface waters”. The draft version 4.0 of the Guidance Chapter R.7b Endpoint specific guidance [16] states that “New simulation studies should be conducted at environmentally relevant temperatures namely at 12 °C as this is seen as the average surface water temperature for the European Union (9 °C for sea water). If information on degradation half-life is already available from existing simulation degradation tests performed at a higher temperature, they should be normalised to a half-life corresponding to 12 °C by using the Arrhenius equation”. A generic activation energy of 65.4 kJ mol^−1^ is proposed which has been derived by EFSA [19] (see below). The draft version 3.0 of the Guidance Chapter R.11 PBT/vPvB assessment [17] refers to this statement. Simulation degradation studies should be performed according to the OECD guidelines no. 307 for aerobic degradation in soil [37], no. 308 for anaerobic and aerobic transformation in water and sediment [38] and no. 309 for aerobic mineralisation in surface water [39]. However, Honti and Fenner [30] concluded that available OECD 308 data are insufficient to derive persistence indicators that had both acceptable robustness and uncertainty. Rauert et al. [42] proposed “…normalising DegT50 values to 12 °C because this temperature is established or suggested under the majority of frameworks (i.e. Biocides Regulation, REACH and medicinal products Directives)”. However, the guidance for PBT/vPvB assessment is the same for the three mentioned frameworks. For PPP, DG [10] stated in its Working Document on “Evidence needed to identify POP, PBT and vPvB Properties for Pesticides”: “Laboratory studies: DT50 values should be normalised to a temperature of 20 °C, as this is the current practice in recent assessments of soil degradation rates of active substances”. This Working Document has been applied for the initial establishment of the list of candidates for substitution [11] as required in Article 80 [7] of Regulation (EC) No 1107/2009 [24], i.e. half-lives at 20 °C have been considered when the list was compiled.

### QSAR approaches

Simulation studies are preferred for the determination of biodegradation half-lives. However, for the vast majority of industrial chemicals no data are available from simulation studies. Thus, information from test on ready and inherent biodegradability as well as from (Q)SAR methods (BIOWIN from EPI Suite, [54]) has been recommended for screening substances as being potentially persistent [15]. The screening criteria, laid down in Table R.11-4, are not legally binding whereas the (definitive) P/vP criteria are (see Table 1). Testing on biodegradability is conducted at room temperature and not temperature corrected. BIOWIN generates biodegradation half-lives in water and assigns them to five classes representing half-lives from hours to years. The half-life in water is multiplied by factors of 2 and 9 to obtain a half-life in soil and sediment, respectively. BIOWIN models for primary and ultimate degradation have been developed from expert surveys [6]. Arnot et al. [3] calibrated the models to empirical aerobic environmental half-life data from 40 selected “training set” chemicals and evaluated the outcome of this calibration with environmental aerobic biodegradation data of a set of 115 chemicals. Although it is not explicitly stated at which temperature biodegradation half-lives were determined, room temperature for laboratory and summer temperature for field measurements can be assumed. Regression analysis was undertaken to identify statistical relationships between biodegradability and basic properties. As the QSAR models were trained using data on biodegradation half-lives at room temperature, any predictions will also refer to this temperature. Temperature correction of the results of biodegradability testing and BIOWIN calculations is not required for screening information [15].

### Overall persistence

Persistence criteria are based on the hazardous properties of hydrophobic neutral reference chemicals. Under this approach, it is assumed that the objective of the P assessment is to avoid chemicals similar to known PBTs such as PCBs. These known PBTs have different half-lives in different media. Degradation in water is assumed to be faster than in soil and sediment. An alternative approach, called management approach [7, 43] is to attempt to limit the overall residence time of a chemical in the environment as the goal of the assessment scheme. The overall residence time or the reciprocal overall persistence, *P*_ov_, can be calculated by a multimedia model, which takes into account environmental degradation as well as distribution [33, 44]. The objective is to control the presence of chemicals in the environment and not to compare half-lives against a pre-defined set of media-specific criteria. Thus, average environmental temperature could be considered in multimedia modelling as it is done for calculation of PEC_regional_ [15].

## Accumulation in the environment

The main concern underlying the persistence assessment is resistance to degradation processes and accumulation in soil, water or sediment causing unexpected long-term effects on living organisms. The regulatory P-criteria must ensure that this concern does not materialise, with a sufficient margin of safety.

**Fig. 1:**
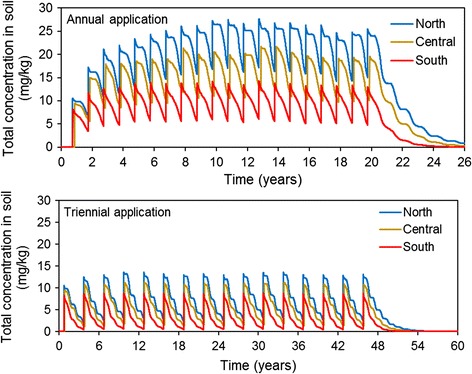
Concentration of a PPP with a half-life of 120 days at 20 °C in soil calculated for the EFSA soil scenarios in the Northern, Central and Southern Regulatory Zones [18, 20] for annual (*top panel*) and triennial application (*bottom panel*)

This was explored for a reference temperature of 20 °C assuming *realistic worst case* soil/climate/crop scenarios in the EU developed by EFSA [18]. The average annual air temperature for the scenarios relevant to total soil in the Northern, Central and Southern European Zone is 4.7, 8.0 and 11.0 °C, respectively. A PPP with a half-life of 120 days at 20 °C was assumed to be applied at each location to a winter cereal crop every year for 20 years, or every 3 years for 45 years. Triennial application is common for PPP as these are often specific to certain agricultural crops, and these crops are grown in a rotation with other crops which are treated with different products. Concentrations of the PPP in soil were calculated using the FOCUS model PELMO [32], an exposure model that is well accepted in the regulatory framework [27] and which considers extrapolation of the degradation rate constants to the respective temperature values in the soil on a daily basis. The scenario parameterisation for this model is provided by EFSA [20]. The climate conditions reflect actual conditions over multiple years at the scenario sites and change throughout the simulation period in order to account for the natural variability between different years at the same location. Figure 1 shows that the rate of decline in concentrations after application differs between the various years, and even within a single year as a result of the temporal variation in the climate. The calculations were continued for a number of years after the last application.

Figure 1 (upper panel) shows that the maximum and minimum simulated total concentration in soil reached just after an application initially increases year-on-year, because there is some residual chemical left from the previous application the year before. It is a mathematical consequence of first-order degradation that the minimum and maximum concentrations stabilise at a certain level over time, if the frequency and magnitude of the emission remain constant. The levels, and the time at which stabilisation occurs, depend on the half-life and the actual environmental conditions. Figure 1 (upper panel) shows the pattern of concentrations that can be expected for *realistic worst case* soil/climate/crop scenarios in the EU based on the half-life of 120 days in soil at 20 °C, considering the temporal and spatial variability. The build-up of concentrations in soil stabilises after 6, 8 and 10 years in the Southern, Central and Northern zone, respectively. Thus, there is a range of 8 ± 2 years, i.e. the variability in the time after which the plateau is reached in the three zones is 25% (coefficient of variation, CV). The simulated minimum residues before the next application stabilise at around 6 mg/kg soil in the Southern climate zone, 10 mg/kg soil in the Central zone and 16 mg/kg in the Northern zone, this corresponds to a CV of 47%. The CV of the simulated maximum concentrations (approximately 13, 20 and 27 mg/kg soil in the three climate zones) is 35%. These CVs reflect the spatial variability between the three zones.

The results in Fig. 1 (lower panel) show that the residues stabilise at much smaller levels if an application is only made every 3 years. Moreover, Fig. 1 (lower panel) shows that there is a negligible increase of the plateau over time after triennial application in all zones from year 2. The substance is only present for a relatively short time after the last release; the concentrations in soil decline steadily to negligible levels within a few years.

**Fig. 2:**
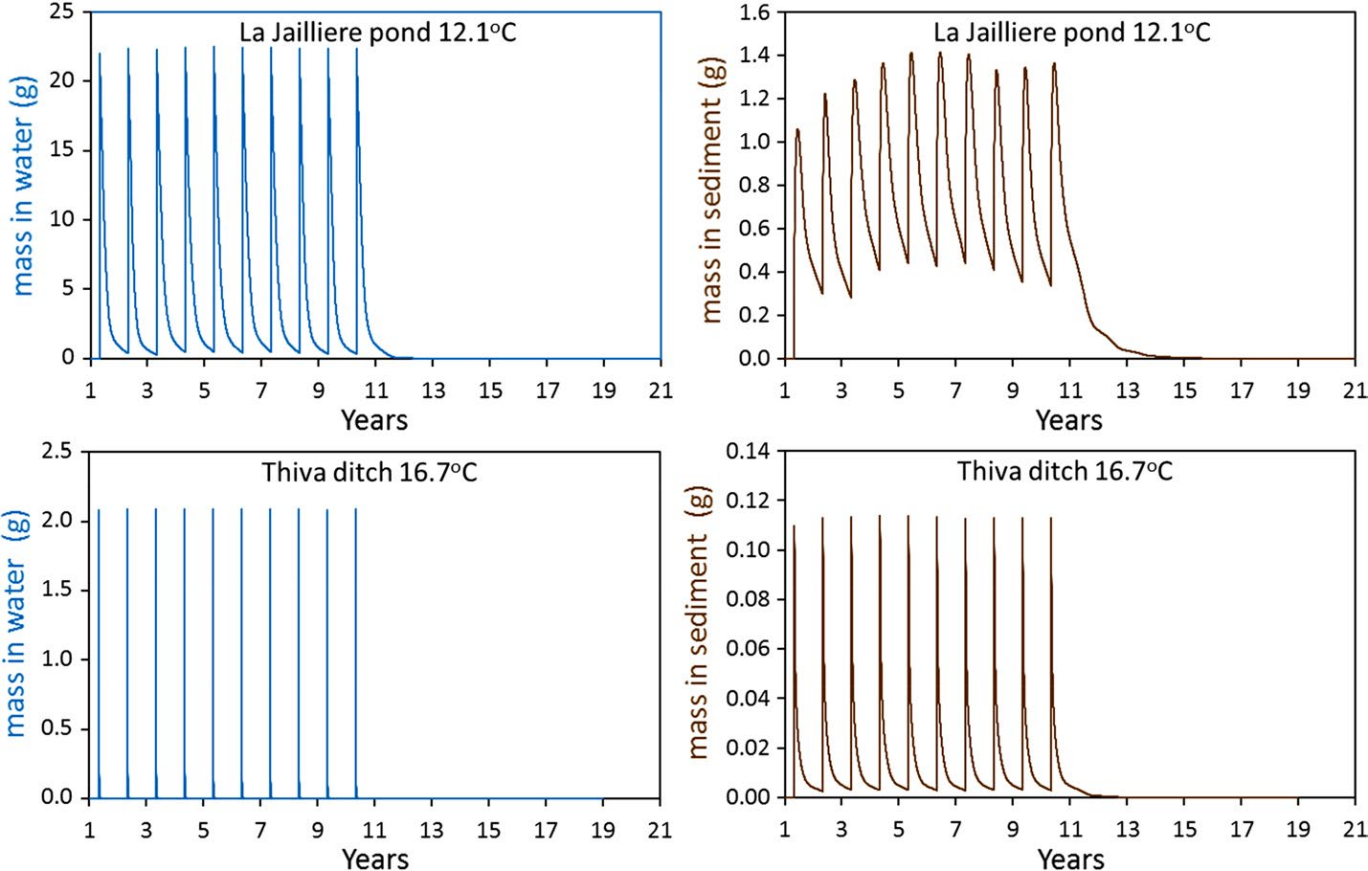
Mass of a PPP with a half-life of 40 days in water and 120 days in sediment at 20 °C calculated for the FOCUS surface water scenarios La Jailliere (*top panels*) and Thiva (*bottom panels*) for water (*left panels*) and sediment (*right panels*)

Additional calculations were undertaken for a hypothetical compound discharged to EU surface waters (Fig. 2). The chemical has a half-life for fresh water of 40 days and for sediment of 120 days at 20 °C. The FOCUS surface water model TOXSWA was used to calculate the mass of the chemical in water and sediment of a pond and a ditch for two of the FOCUS locations with annual average temperatures of 12.1 and 16.7 °C [29]. The model adjusts the half-life to the actual daily temperature of the scenario. The chemical was discharged into the water once a year for 10 years with variable climate conditions. Figure 2 shows that even in a static pond there is no long-term increase of the annual maximum mass in the water, very limited increase in the sediment over the 10 years and the chemical disappears within a few years of the last emission.

### Experimental temperature to determine degradation half-lives

The purpose of simulation degradation testing in the context of the P-classification is to determine a half-life for comparison with the persistence criteria defined by the EU regulations. In principle, tests could be undertaken at any temperature within the ranges given in the test guidelines and the resulting half-lives could be adjusted to other temperatures using correction procedures. However, this extrapolation carries some uncertainties. It is therefore advantageous to undertake the test at the actual temperature of interest (20 °C is appropriate for the P-criterion) and avoid the need for extrapolation. There are additional advantages of a test temperature of 20 °C and these are discussed below.

### Practical considerations and comparability with existing data

Historically, degradation tests for PPP regulatory purposes have been undertaken at 20 °C, with few studies at 10 °C. This is partly for practical reasons. There is a much wider availability of laboratory test facilities to study degradation at 20 °C than at lower temperatures, less risk of study temperatures to deviate from the target, and the costs are reduced. There is now a large database of experimental half-lives at 20 °C (e.g. The University of Hertfordshire Pesticide Properties Database, http://sitem.herts.ac.uk/aeru/ppdb/en/index.htm). This allows benchmarking of results for new chemicals against the existing data.

### Uncertainties in the half-lives

**Fig. 3:**
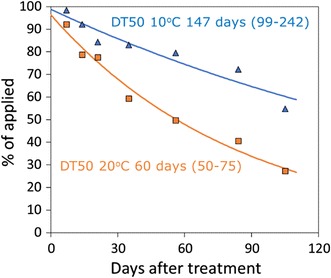
Measured residues of a PPP in soil at two temperatures (*symbols*) and optimised decline curves (*lines*), with 95% confidence intervals of the optimised half-life in *brackets*

Half-lives are derived by fitting kinetic models to chemical residues plotted against time. The FOCUS work group on degradation kinetics [28] gives guidance on how to undertake the model fitting. The group stressed that the data set must be of sufficient quality to clearly establish the dissipation pattern. It is difficult to obtain robust kinetic fits to data that decline slowly over time. In these cases, the confidence intervals of the estimated degradation rate constants are often large, indicating parameter uncertainty. This is because there is not enough information inherent in the data to obtain a reliable estimate of degradation. At low temperatures, the study period is simply too short to give a sufficient decline in the residues beyond the half-life. A test temperature of 20 °C causes a faster decline of the residues and allows a more reliable kinetic analysis. This is illustrated in Fig. 3 using hypothetical data. The graph shows an example fit of first-order kinetics to the mass of a chemical measured at 10 and 20 °C. The 95% confidence interval of the half-life at 10 °C is 99–242 days, this is much wider than the confidence interval at 20 °C of 50–75 days.

For a robust assessment of the degradation rate constant, the residues should ideally decline to 10% by the end of the study, but at least reach the half-life (i.e. 50% of initial mass or less). The typical study period of 120 days is long enough to reach the half-life in soil of non-persistent substances at a test temperature of 20 °C. If 12 °C or even 9 °C was used as the reference temperature for the P-criterion, then the P-criterion for soil would have to be much longer. The experimental period could be extended, but this might lead to an undesirable loss of biological activity and additional costs. It is thus much more straightforward to discriminate between persistent and non-persistent substances at a test temperature of 20 °C. Uncertainties in the optimisation could lead to an under-estimation of the half-life and this could result in an incorrect classification of a substance as non-persistent when it is in fact persistent, or vice versa. Where a geometric mean or other form of average is calculated from several half-lives of the same substance, it is important that the individual estimates are accurate because the uncertainty of each estimate is propagated into a greater uncertainty of the average. As it is easier to obtain robust half-lives at 20 °C, this is the preferred experimental temperature.

### Identification and quantification of metabolites

At higher temperatures, metabolites are formed at larger quantities and earlier in the study period. Breakdown products that are classified as major metabolites at 20 °C may not be detected at all at 10 °C. Deriving robust half-lives for metabolites at 10 °C will often be impossible. Performing the test at 20 °C will avoid having to carry out two experiments, one to derive the half-life of the parent compound and one to detect and identify metabolites. This will not only save experimental effort, but also time for kinetic analysis and reporting, regulatory evaluation and decision making.

## Extrapolation to other temperatures

The relationship between the degradation rate constant and temperature is often described by the Arrhenius or similar equations [19]. The Arrhenius equation assumes that the first-order rate constant of degradation depends on the activation energy *E*_*a*_ of the reaction and the temperature at which the reaction occurs.

$$k = A\, exp^{{ - \left( {\frac{Ea}{R T}} \right)}}.$$

*k* = degradation rate constant [d^−1^]. *A* = factor equal to the rate coefficient at infinite temperature [d^−1^]. *E*_*a*_ = activation energy [kJ mol^−1^]. *R* = gas constant = 0.008314 [kJ mol^−1^ K^−1^] and *T* = absolute temperature [K].

The degradation half-life is related to the degradation rate constant by ln (2)/*k*. The experimental half-life can be extrapolated to the temperature of interest using this relationship:

$$t_{1/2} \left( {T\text{ref}} \right) = t_{1/2} \left( T \right) \text{exp}^{{\left( {\frac{Ea}{R}\left[ {\frac{1}{Tref} - \frac{1}{T}} \right]} \right)}}$$

*t* ½ *(T)* = half-life at experimental temperature *T* [days] and *t* ½ *(Tref)* = half-life at temperature of interest *Tref* [days].

The equations could in principle be used to adjust a half-life measured at 20 °C in a degradation simulation test to a half-life at any other temperature, for example 12 °C. ECHA [15, 16] guidance recommends a simplified version of the equation shown here to correct from the experimental temperature to 12 °C:

$$t_{1/2} \left( {12\; {^\circ}\text{C}} \right) = t_{1/2} \left( {T \;{^\circ}\text{C}} \right) exp^{{\left( {0.08 \left [ {T\; {^\circ}\text{C} - 12\; {^\circ}\text{C}} \right]} \right)}}.$$

The validity of the Arrhenius equation has been discussed extensively. It is based on the concept that a certain amount of energy (i.e. the activation energy) is needed for a chemical reaction to occur. In surface waters, sediment and soils, chemical breakdown is mediated by a number of different reactions, including cleavage of molecules by enzymes released by micro-organisms, photolytic and hydrolytic reactions. These reactions will not all have the same activation energy. Microbial transformations further depend on the abundance and activity of micro-organisms and the stability of enzymes which are also related to temperature. The temperature at which micro-organism is most active can vary from species to species.

The composition of the microbial population is not the same in all surface waters, sediments and soils. The potential for microbial degradation of chemicals will, therefore, also vary between biological systems. The interesting question is: even if the various systems have a different absolute level of microbial activity, is the relative change of this activity as a result of changing temperature the same in all systems? This question can be split into two aspects: (i) is the shape of the temperature response (linear, exponential, etc.) the same in all systems, i.e. can the same type of equation be used to describe this response, and (ii) are the parameters of the response curve the same in all systems? In the context of this opinion paper, this translates to the following: (i) is the Arrhenius equation valid in all systems, and (ii) is the activation energy the same?

For PPP, there is a wealth of experience with the use of the Arrhenius equation from the research and regulatory context. There is a large body of evidence that degradation in the field can be well predicted based on the Arrhenius equation if the difference in temperature between field and laboratory is taken into account. EFSA [19] collated a large number of degradation studies for PPP in soil conducted at different temperatures and fitted the Arrhenius equation to the half-lives. They concluded that the Arrhenius equation is valid over the temperature range from 0 to 30 °C. This was based on an analysis of 56 datasets. The frequency distribution of activation energies from 99 datasets had a median of 65.4 kJ mol^−1^. This activation energy was recommended by EFSA as a generic value for the temperature extrapolation within the exposure assessment for PPP. However, it was acknowledged that the activation energy for the temperature response of PPP degradation can vary from compound to compound. Variations in temperature response parameters have also been found by Bagi [4] for hydrocarbon biodegradation in marine environments. Alidina et al. [2] observed that the temperature response of chemical attenuation of trace organic chemicals in managed aquifer recharge systems depended on the compound.

In conclusion, there are uncertainties in the Arrhenius equation, and in the use of generic activation energy for all substances and media. It is therefore preferred to measure degradation at the temperature of interest. Where extrapolation is necessary, the option which introduces the least uncertainty into the decision making is preferred. For industrial chemicals and biocides, we have two options regarding the regulatory assessment: (i) measure the half-life at 20 °C and use this for comparison with the P-criterion, extrapolate to 12 °C for the risk assessment, or (ii) measure at 12 °C and use this half-life in the risk assessment, extrapolate to 20 °C for comparison with the P-criterion. Option (i) would lead to a more accurate half-life for the persistence assessment. There would be some uncertainty in the risk assessment for industrial chemicals and biocidal products, but this uncertainty is overruled by the many other uncertainties inherently contained in exposure modelling. Moreover, the impact of degradation half-lives on exposure modelling outcome, i.e. PECs, is less relevant than for the P-assessment (see above). Option (ii) on the other hand places the uncertainty into the half-life used for comparison with the P-criterion. This has a much greater impact on the decision making particularly for PPP where the exceedance of the P-criteria could mean that there is no further assessment and the product cannot be registered. Therefore, on balance, it is preferred to measure at 20 °C and extrapolate to 12 °C if necessary. In the exposure assessment for PPP, an extrapolation is undertaken from the temperature at which the simulation tests are undertaken to the actual daily temperature in multi-year modelling scenarios for various European locations. Here, the uncertainty will be greater if the half-life is extrapolated to temperatures far above or below the reference temperature. A measurement temperature of 20 °C appears to be a good compromise.

To our opinion, undertaking biodegradation tests at 20 °C and extrapolating to different environmental temperatures where necessary seems to be the best option, as long as the limitations of this approach are kept into consideration. Temperature should be systematically varied in laboratory incubation studies to better understand its impact on the biotransformations of chemicals.

## Conclusions

There is a contentious issue in EU regulatory frameworks at which experimental temperature degradation simulation tests should be undertaken, and which temperature is relevant for comparison with persistence criteria. Two main alternatives are being considered: 20 °C and a lower temperature (i.e. 9 °C for marine water and 12 °C for all other systems). There is a lot of experience gained from soil and water/sediment simulation tests conducted at 20 °C (or room temperature), in particular for PPP, but only few data from testing at lower temperatures. This opinion comes to the conclusion that measuring degradation at 20 °C has several advantages over lower temperatures in the light of accuracy and uncertainty as well as the regulatory purpose:

1. Concentrations of metabolites/transformation products are higher and thus are identified with higher accuracy.
2. Robust kinetic fits of dissipation processes are better achieved for higher temperature with less uncertainty.
3. Comparison of degradation half-lives with P/vP-criteria at 20 °C is in line with the intentions of some international agreements on PBT chemicals and POPs. Scenario calculations for PPP with half-lives of 120 days at 20 °C in soil and sediment, and of 40 days in water, adjusted to various European climate zones, demonstrate the expected concentration patterns. But the relevant temperature for comparison with persistence criteria is a regulatory question that needs a broad evaluation of the possible solutions and their relative advantages and disadvantages, based on scientific background and political intentions of the regulatory frameworks.
4. Uncertainty of persistence classification is smallest when measured degradation half-lives are used for direct comparison with P/vP assessment criteria.
5. Extrapolation of half-lives to lower temperatures using the Arrhenius equation is justified for assessing exposure to industrial chemicals and biocidal products in a standard European environment as well as for comparing degradation of PPP in different European climate zones as long as the limitations of this approach are kept into consideration.
6. Additional systematic laboratory studies focusing on the temperature range below 20 °C in soils, and water–sediment systems for a range of contaminant classes would enhance our understanding of the impact of environmental temperature variation on the biotransformations of chemicals.

## Abbreviations

B: bioaccumulative
BCF: bioconcentration factor
CV: coefficient of variation
DT50: time for 50% dissipation or disappearance
FOCUS: Forum for International Co-ordination of pesticide fate models and their Use
ISO: International Standards Organization
LRT: long-range transport
OECD: Organisation for Economic Cooperation and Development
OSPAR: Oslo Paris Convention
PEC: predicted environmental concentration
P: persistent
PNEC: predicted no-effect concentration
PBT: persistent, bioaccumulative, and toxic
POP: persistent organic pollutant
P_OV_: overall persistence in all compartments
PPP: plant protection product
(Q)SAR: (quantitative) structure–activity-relationship
REACH: Registration, Evaluation, Authorization and Restriction of Chemicals
T: toxic
*t*_½_: half-life
TGD: Technical Guidance Document
UNEP: United Nations Environment Program
vB: very bioaccumulative
vP: very persistent

## Footnotes

1 The time for 50% decline in the initial mass by degradation and/or other loss mechanisms is referred to in the literature as DT50, DegT50, half-life or *t*½. Throughout this article, half-life is used and this is abbreviated as *t*½.
